# A cross sectional survey to estimate prevalence and associated factors of asthma on Reunion Island, Indian Ocean

**DOI:** 10.1186/s12889-019-7031-7

**Published:** 2019-05-30

**Authors:** J-L. Solet, C. Raherison-Semjen, E. Mariotti, Y. Le Strat, A. Gallay, E. Bertrand, N. Jahaly, L. Filleul

**Affiliations:** 1 Santé Publique France [The French Public Health Agency], Indian Ocean Regional Office, Saint-Denis, Reunion France; 2 Inserm U219, Institute of Public Health, Epidemiology, and Development (ISPED), Bordeaux University, Bordeaux, France; 3 Agence de santé océan Indien [Indian Ocean Health Agency], Saint-Denis, Reunion France; 4 Santé publique France, [The French Public Health Agency], Saint-Maurice, France; 5 Bureau d’études Synthèses [Synthesis analysis office], Saint-Denis, Réunion France; 6 Syntheses Mauritius LTD, Quatre Bornes, Mauritius

**Keywords:** Asthma, Epidemiology, Prevalence, Reunion, Indian Ocean

## Abstract

**Background:**

Previous studies on asthma mortality and hospitalizations in Reunion Island indicate that this French territory is particularly affected by this pathology. Epidemiological studies conducted in schools also show higher prevalence rates in Reunion than in Mainland France. However, no estimates are provided on the prevalence of asthma among adults. In 2016, a cross-sectional survey was conducted to estimate the prevalence of asthma and to identify its associated factors in the adult population of Reunion Island.

**Methods:**

A random sample of 2419 individuals, aged 18–44 years, was interviewed by telephone using a standardized, nationally validated questionnaire. Information was collected on the respiratory symptoms, description of asthma attacks and triggering factors for declared asthmatics, as well as data on the indoor and outdoor home environment. “Current asthma” was defined as an individual declaring, at the time of the survey, having already suffered from asthma at some point during his/her life, whose asthma was confirmed by a doctor, and who had experienced an asthma attack in the last 12 months or had been treated for asthma in the last 12 months. “Current suspected asthma” was defined as an individual presenting, in the 12 months preceding the study, groups of symptoms suggestive of asthma consistent with the literature.

**Results:**

The estimated prevalence of asthma was 5.4% [4.3–6.5]. After adjustment, women, obesity, a family member with asthma, tenure in current residence and presence of indoor home heating were associated with asthma. The prevalence of symptoms suggestive of asthma was 12.0% [10.2–13.8]. After adjustment, marital status, passive smoking, use of insecticide sprays, presence of mold in the home and external sources of atmospheric nuisance were associated with the prevalence of suspected asthma.

**Conclusion:**

Preventive actions including asthma diagnosis, promotion of individual measures to reduce risk exposure as well as the development of study to improve knowledge on indoor air allergens are recommended.

## Background

The World Health Organisation (WHO) estimates that 235 million people around the world have asthma and that it led to 255,000 deaths in 2005 [1]. In Europe, the prevalence of asthma decreases from north to south. The United Kingdom and Ireland are particularly affected, with prevalences surpassing 30%. France falls into the middle of the range [2]. However, prevalences in the French overseas departments exceed the national average [3]. Reunion, a French overseas department, an island in the Indian Ocean with an area of 2512-km2 and a population of 850,996 (2016 population estimate based on the French National Institute of Statistics and Economic Studies (INSEE) census). Previous studies have indicated that the area is particularly affected by asthma. Adjusting for age and gender, asthma mortality rates are 3 to 5 times higher than in mainland France and hospitalisations for asthma are twice as common [4–6]. School-based studies have shown that Reunion is one of the regions with the highest prevalences among both young children and adolescents [7, 8].

However, information on the prevalence of asthma among the general adult population has been lacking. For this reason, a cross sectional survey was conducted in 2016 to estimate the prevalence of asthma in the general population of adults aged between 18 and 44 years living in Reunion. The secondary objectives of the study were to describe the characteristics of asthmatic patients and of individuals with symptoms suggestive of asthma, and to study how the domestic environment affects the risk of asthma in order to improve prevention strategies, particularly individual-level measures.

## Methods

### Study population

Between 8th February and 1st June 2016 a phone survey was conducted amongst a random and representative sample of individuals aged between 18 and 44 years. People under the age of 18 were excluded because there was already data available on asthma in children and adolescents in Reunion [7, 8]. People over 44 years were excluded in order to reduce the risk of misclassification of asthma symptoms as those of chronic obstructive pulmonary disease (COPD), which is more common among older smokers. The questionnaire primarily included questions from the European Community Respiratory Health Survey (ECRHS) [9] and the Epidemiological study on the Genetics and Environment of Asthma (EGEA) [10]. The questionnaire was developed in conjunction with respiratory and allergy medical specialists in Reunion.

The target population was stratified into two groups. The first group consisted of individuals who resided in households equipped with a landline telephone, while the second group was composed of individuals residing in households equipped with a mobile phone but no landline telephone (the “only mobile” group). The proportion of interviewees from each group (70% from the landline group and 30% from the only mobile group) was determined based on reference statistics [11]. Sampling involved dialling randomly generated telephone numbers in order to include people not listed in the phone book for the landline group and clients from the different local mobile operators for the mobile phone group. Telephone numbers that did not lead to an interview (e.g. due to non-response, refusal, unavailability, inaccessibility, non-eligibility) were replaced by successive incrementing. The replacement of an unsuccessful number due to non-response only occurred after repeated calls, set at a minimum of 8 attempts, at different times of the day and days of the week.

In responding households with more than one eligible household member, the household composition was recorded, and a random draw undertaken to select the person to be interviewed. The questionnaires were administered in French and Creole by trained investigators, using ASKIA software, and the CATI (computer-assisted telephone interview) system. The questionnaire included questions about sociodemographics (e.g. gender, age, commune of residence, level of education, and place of birth), anthropometrics (e.g. height, weight, and body mass index (BMI)), smoking habits, chronic health problems and diseases, symptoms suggestive of asthma, asthma attacks and trigger factors, and the interviewees’ home environment (both interior and exterior). In order to reduce the reporting bias, asthma was not mentioned at the beginning of the interview. Investigators introduced the questionnaire as a general survey on health and the home environment. All questions relating to symptoms were asked prior to the investigators mentioning asthma itself.

#### Case definitions

- Current asthma: any person reporting ever receiving a diagnosis of asthma from a doctor, and who had experienced an asthma attack or was treated for asthma in the preceding 12 months.

- Suspected current asthma: Any person reporting one or more groups of symptoms suggestive of asthma consistent with the literature in the preceding 12 months (wheezing, resting, nocturnal and exertion dyspnea, nocturnal respiratory discomfort, nocturnal cough) [12] but not classified as asthmatic according to the definition of current asthma.

#### Data analysis

The estimators produced were consistent with the sampling design (stratification, sampling weights, primary sampling units). Additionally, post-stratification weighting was implemented, using the “Reweight Iterative Method”, to adjust estimates to the population age and gender distribution obtained from the INSEE 2013 census of the population. These estimates were calculated along with their 95% confidence intervals (CI) taking into account the adjustment made to the sample and its impact on increasing the variance of the proportion estimator. Chi-square tests were used to compare proportions between groups. Univariate analysis was used to calculate odds ratios and their 95% CI estimated by the Woolf method.

Data were analysed using Xlstat and R software. Multivariate analysis using logistic regression models was undertaken to identify the factors associated with current asthma and suspected asthma.

In each model, a pre-selection of explanatory variables was conducted by reviewing all variables considered as risk factors for asthma (e.g. gender, age, smoking, BMI, having a family member with asthma) as well as variables that could potentially be associated with asthma (such as the home environment). At the end, 23 explanatory variables were chosen to be tested in each model. The glmulti library in the R software was then used for an initial selection of variables through the likelihood ratio test and the Akaike information criterion. The first order interactions were then added to the initial models and an exhaustive selection of models was conducted with the glumult algorithm.

#### Ethics

At the time of the survey, French regulations did not require the approval of an ethics committee for observational studies that did not include performing acts or using products on the participants, as was the case for this study. The study protocol was approved by an internal expert committee (CCEP) of the French Public Health Agency. In addition, a declaration was made to the National Commission on Informatics and Freedoms (CNIL) in accordance with the Data Protection Act.

Contacted households and selected participants were informed that participation was optional and about their rights of access, rectification and opposition to the data concerning them.

## Results

The final sample consisted of 2419 individuals, including 1706 individuals in the “landline” group and 713 individuals in the “only mobile” group. Achieving these required more than 4700 call hours and more than 208,000 telephone calls (including false numbers, non-responses, ineligibility, refusals). A total of 6939 interviews were started, i.e. the phone was answered and information on household composition collected. The cessation of 4338 interviews was mainly due to two reasons: the absence of persons aged 18–44 in the household (2855 cases) and the selection of a participant not available at the time of the call (1271 cases). The interview was ceased due to refusal to participate in 212 calls. In addition, 182 interviews were ceased during the interview following the participant’s refusal to continue. These incomplete questionnaires were not included in the analysis. Amongst valid questionnaires, the response rate to individual questions was high (greater than 95%). Throughout the survey, quality checks of the interviews were regularly carried out by the supervisor responsible for monitoring the study.

The average age of participants was 34 years old [18 to 44 years old]. The sample was comprised of 47.3% men and 52.7% women, with a gender ratio of 0.81 (Table 1).

**Table 1:** Sociodemographic profile of respondents, Reunion, 2016

	N observed	% estimated	95% CI
Gender			
Male	1080	47.3%	[45.0%; 49.6%]
Female	1339	52.7%	[50.4%; 55.0%]
Age groups			
18–19 years	138	8.3%	[7.0%; 9.6%]
20–24 years	401	18.1%	[16.3%; 19.9%]
25–29 years	301	17.3%	[15.5%; 19.1%]
30–34 years	369	17.2%	[15.4%; 19.0%]
35–39 years	460	18.7%	[16.9%; 20.5%]
40–44 years	750	20.4%	[18.5%; 22.3%]
Occupation-status			
Actively employed (salaried or otherwise)	1271	48.4%	[46.1%; 50.7%]
Actively unemployed	521	22.7%	[20.7%; 24.7%]
Never worked (inactive)	179	9.4%	[8.0%; 10.8%]
Other inactive (retirees…)	448	19.5%	[17.6%; 21.4%]
Socio-professional categories			
Clerks	747	29.6%	[27.5%; 31.7%]
Laborers	419	18.6%	[16.8%; 20.4%]
Intermediate occupations	368	13.7%	[12,1%; 15,3%]
Senior managers, liberal professions	170	5.6%	[4.5%; 6.7%]
Artisans, traders, company managers	64	2.6%	[27.5%; 31.7%]
Farmers	24	1.0%	[0.5%; 1.5%]
Others and inactives	627	28.8%	[26.7%; 30.9%]
Marital status			
Single	1256	56.2%	[53.9%; 58.5%]
Married or civil partnership	674	23.1%	[21.1%; 25.1%]
Not married but cohabiting	394	17.9%	[16.1%; 19.7%]
Divorced	74	2.2%	[1.5%; 2.9%]
Widower	10	0.3%	[0.0%; 0.6%]
Unspecified	11	0.5%	[0.2%; 0.8%]
Place of birth			
Reunion	1914	79.6%	[77.7%; 81.5%]
Mainland France	370	14.4%	[12.8%; 16.0%]
Mayotte	39	2.5%	[1.8%; 3.2%]
Madagascar	38	1.4%	[0.9%; 1.9%]
Mauritius	19	0.6%	[0.2%; 1.0%]
Other	39	1.5%	[0.9%; 2.1%]
Length of time in Reunion			
Less than 5 years	153	7.0%	[5.8%; 8.2%]
Between 5 and 10 years	107	4.4%	[3.4%; 5.4%]
Between 10 and 15 years	138	4.5%	[3.5%; 5.5%]
Between 15 and 30 years	895	43.8%	[41.5%; 46.1%]
More than 30 years	1125	40.2%	[37.9%; 42.5%]
Unspecified	1	0.0%	–
Total	2419	100%	

### Prevalence of current asthma

The estimated prevalence of current asthma was 5.4% [4.3–6.5] among the population of individuals aged between 18 and 44 years living in Reunion (Table 2).

**Table 2:** Observed prevalence and estimation of the current prevalence of asthma, Reunion, 2016

	Population observed	% observed	% estimated	CI 95%
Current asthma^(a)^	133	5.5	5.4	4.3–6.5
Overall	2419	100.0	100.0	

^(a)^Over the last 12 months

The prevalence was higher in women (7.0%; 95% CI: 2.2–4.8) than in men (3.5%; 95% CI: 5.4–8.6) (*p* < 0.0001) across all age groups. Women aged between 18 and 24 years were most affected, with a prevalence of 9.8% (95% CI: 5.8–13.8).

The prevalence of current asthma did not vary significantly according to the occupational status, socio-professional category, level of education, place of birth and/or the number of years the person had lived on Reunion Island, or the place of employment. Obesity (defined as a BMI greater than or equal to 30 kg/m^2^) was related to asthma, with a prevalence of 8.3% (95% CI: 4.7–11.9) versus 5.4% (95% CI: 4.0–6.8) among people with a normal body mass (a BMI of 18.5 to 24.9 kg/m^2^). (Odd Ratio (OR): 1.59; 95% CI: 1.00–2.52). Those with an immediate family member diagnosed with asthma had a higher prevalence of asthma of 8.3% (95% CI: 5.9–10.1) compared to a prevalence rate of 3.6% for those without a family history (95% CI: 2.5–4.7), OR: 2.33 (95% CI: 1.62–3.35).

Regarding domestic environmental factors, univariate analyses showed that the prevalence of asthma was higher amongst those living in their current residence for less than 5 years (OR: 3.56; 95% CI: 1.77–7.64). The prevalence was significantly higher when there was a reported source of moisture in immediate proximity to the residence (less than 10 m) (OR: 1.71; 95% CI: 1.18–2.47). Similarly, having obstructed air vents in the home was associated with a higher prevalence (OR: 3.95; 95% CI: 1.72–9.06). The presence of mould, moist spots, or fungus in the home was associated with a higher prevalence of asthma (OR: 1.54; 95% CI: 1.08–2.19). The permanent presence of cockroaches (defined as sightings every, or almost every day) was also associated with a higher prevalence (OR: 2.33; 95% CI: 1.27–4.28).

The results of the multivariate analysis adjusted by age are shown in Table 3.

**Table 3:** Risk factors for current asthma (multivariate), Reunion, 2016

Variables		N	Prevalence	Ora^(a)^	*P*-value	95% CI
Gender	Male	1080	3.5%	1.00		
	Female	1339	7.0%	1.97	3.62 10^−4^	[1.32; 2.96]
Family member with asthnma	No	1478	3.7%	1.00		
	Yes	917	8.1%	2.29	5.25 10^− 6^	[1.53; 3.42]
Length of time at curent residence	Less than 5 years	1090	7.2%	3.51	1.13 10^− 3^	[2.35; 5.25]
	5 to 9 years	458	4.8%	2.35	2.58 10^−3^	[1.45; 3.79]
	10 to 19 years	502	4.4%	2.04	2.80 10^−2^	[1.26; 3.31]
	20 years or more	369	2.0%	1.00		
Domestic heating	No	2222	5.2%	1.00		
	Yes	197	8.1%	1.79	3.60 10^−2^	[1.04; 3.09]

^(a)^ Adjusted odds ratio

After adjustment, the prevalence of asthma was higher among women than men and among people who reported having family members with asthma and those living in homes with heating. However, the prevalence decreased with length of time in the current home.

### Prevalence of suspected asthma

The prevalence of persons who reported experiencing the symptoms suggestive of asthma in the last 12 months was estimated to be of 12.0% (95% CI: 10.2–13.8%). Among these, 8.8% did not meet the definition of current asthma. This is important because it suggests that a large percentage of people with symptoms suggestive of asthma have not been diagnosed. In addition, 2.2% of those who met the definition of current asthma did not experience any symptoms suggestive of asthma in the last 12 months. Overall, 14.2% (95% CI: 12.3–16.1) of all people aged between 18 and 44 years reported having current asthma or the symptoms suggestive of asthma.

The prevalence of suspected current asthma was slightly higher in women (9.7%; 95% CI: 7.8–11.6) than in men (7.9%; 95% CI: 6.0–9.8), though the difference was not statistically significant (*p* = 0.120). Univariate analysis showed that the prevalence of suspected current asthma did not vary significantly according to occupational status, socio-professional category, level of education, place of birth and/or the number of years the person has been living in Reunion, or the place of residence or employment. The main risk factors for suspected asthma were obesity (OR: 1.52; 95% CI: 1.02–2.28), being underweight (OR: 1.76; 95% CI: 1.07–2.92), having an immediate family member with asthma (OR: 1.70; 95% CI: 1.28–2.26), being around regular smokers (OR: 1.34; 95% CI: 1.01–1.78), and marital status, with a higher prevalence amongst single, divorced, or widowed persons than for those who were married, in a civil union, or cohabitating (OR: 1.73; 95% CI: 1.27–2.36).

The results of univariate analyses on environmental factors showed that the presence of mould, moisture, or fungi in the home was associated with a higher prevalence of suspected asthma: 12.0% (95% CI: 9.4–14.6) versus 7.9% (95% CI: 6.3–9.5), (*p* = 0.0015), (OR: 1.58; 95% CI: 1.18–2.10). Similarly, the presence of cockroaches was associated with a higher prevalence of suspected asthma, with the prevalence increasing with the frequency of sightings (Table 4).

**Table 4:** Frequency of cockroach sightings in the home and prevalence of suspected asthma, Réunion, 2016

	Total	Population with suspected asthma	% estimated suspected asthma	95% CI (p)	OR	95% CI (OR)
Throughout the year	428	55	14.1%	[10.2%; 18.0%]	1.94	[1.21; 3.11]
For 2 to 3 months a year	387	34	8.9%	[5.6%; 12.2%]	1.16	[0.69; 1.96]
A few times per year	1007	87	8.4%	[6.4%; 10.4%]	1.09	[0.70; 1.70]
Less than once a year	174	15	6.8%	[2.4%; 11.2%]	0.87	[0.43; 1.75]
Never	290	25	7.9%	[4.3%; 11.5%]	1.00	[0.58; 1.73]
Total	2286	216	9.3%	[7,9%; 10,7%]		

Regular use (defined as every day or almost every day) of insecticide sprays within the residence was also associated with a higher prevalence of suspected asthma: 22.8% (95% CI: 12.6–33.0), (*p* < 0.0001), OR: 3.22 (95% CI: 1.86–5.55) compared to those not using sprays regularly: 8.6% (95% CI: 6.3–10.9).

The results of the multivariate analysis are shown in Table 5.

**Table 5:** Risk factors of suspected asthma (multivariate), Reunion, 2016

Criteria	Answer	N	Prevalence	Ora^(a)^	*P*-value	95% CI
Family member with asthma	No	914	7.4%	1.00		
	Yes	1505	11.5%	1.48	2.22 10^−3^	[1.10; 2.27]
Living close to a source of harmful substances or air pollution	No	1625	6.9%	1.00		
	Yes	794	9.8%	1.33	3.33 10^−2^	[1.10; 2.08]
Presence of smokers in the immediate social circle and mould in the home	Presence of smokers in inner circle and mould in the home	458	14.0%	1.08	4.82 10^−3^	[0.82; 1.62]
	Presence of smokers in inner circle, but no mould in the home	798	7.5%	0.91	7.42 10^−3^	[0.75; 1.59]
	No smokers in inner circle, but mould in the home	387	7.8%	0.60	6.13 10^−3^	[0.37; 1.02]
	No smokers in the inner circle and no mould in the home	776	7.6%	1.00		
Presence of mould in the home and used of insecticide sprays within the home	Insecticide spray and mould in the home	268	10.8%	2.40	9.43 10^−3^	[1.42; 3.62]
	Insecticide spray, but no mould in the home	421	11.6%	2.02	7.51 10^−3^	[1.16; 3.54]
	No insecticide spray, but mould in the home	578	11.4%	2.99	7.73 10^−3^	[1.68; 4.31]
	No insecticide spray and no mould in the home	1152	6.2%	1.00		
Marital Status	Cohabitation	431	6.0%	1.00		
	Married or in a civil union	552	6.7%	1.60	1.81 10^−2^	[0.91; 2.59]
	Single	1366	10.5%	2.30	6.14 10^−2^	[1.37; 3.5]
	Divorced	52	10.6%	5.00	4.95 10^−2^	[1.39; 11.15]
	Widowed	18	15.0%	9.70	4.60 10^−2^	[1.25; 47.09]

^(a)^Odds ratio, adjusted

## Discussion

The prevalence of current asthma amongst 18 to 44 years olds in Reunion, estimated at 5.4% (95% CI: 4.3–6.5), is similar to the previously published prevalence in mainland France of 6.0% (95% CI: 5.7–6.4) amongst individuals over 15 years in a 2003 national health survey [2] and 7.1% amongst those aged over 15 years in the 2012 Institute for Research and Documentation in Health Economics (IRDES) study [13]. Other studies show clear differences in the prevalence of asthma in young children between the two territories [2, 3, 7, 8]. This result raises the hypothesis that the percentage of children on Reunion Island whose asthma has resolved by adolescence is greater than in mainland France. This possibility merits further examination through complementary studies.

However, the case definition for current asthma used in this survey was more restrictive than those used in other national studies since, in addition to an asthma attack and/or treatment during the last 12 months, it included a diagnosis of asthma by a doctor and the criteria of having previously suffered from asthma during the person’s life. Therefore the prevalence in Reunion might have been underestimated due to the more specific case definition.

To compare with the situation in neighboring countries we looked at the results of the world heath survey implemented in 2002–2003 by the WHO in 70 member states. Individuals aged 18 to 45 years responded to questions related to asthma and related symptoms. This study did not use a definition of current asthma close to our but used the following definition for symptoms of asthma: asthma diagnosed by a doctor, and/or a positive response in either of two questions “Have you ever been treated for asthma ”or "Have you been taking any medications or treatment for asthma during the last 2 weeks and/or a positive response to “During the last 12 months have you experienced attacks of wheezing or whistling breath?”. The prevalence of asthma symptoms was 12.85 in Comoros, 12.40 in South Africa and 6.88 in Mauritius [14]. Although the definitions are not similar, it appears that the prevalence of asthma symptoms in Comoros and South Africa are close to the prevalence of suspected asthma in Reunion (12%) but nearly 2 times lower in Mauritius.

We found a higher prevalence of asthma in women than in men, as well as a correlation with obesity, results which have been frequently described in the literature [9, 15–18]. The higher prevalence of asthma among people with a family member with asthma (8.0% versus 3.6%) is consistent with our current knowledge and understanding of the role of genetic factors in the development of asthma [19].

The association between the asthma and the number of years the person has been living in his/her current residence is more complex to understand. The possibility of new furniture being a source of allergens or irritants was suggested. Univariate analysis indicated an association between the presence of moist spots and mould inside the home and the prevalence of asthma (though not a statistically significant factor in the logistic regression model). Many studies have found that traces of moisture in the home are linked to symptoms of asthma, but are also considered as a risk factor for asthma attacks and are suspected of affecting the long-term management of asthma [20–29]. The univariate analysis also revealed that other variables related to the presence of moisture and ambient temperature within the home, such as having obstructed air vents, a heater, or sources of moisture nearby were linked to asthma.

The presence of cockroaches is also associated with a higher prevalence of asthma, and displays a dose-response relationship. This result is consistent with previous studies [30–33].

We also found that suspected asthma shares some common risk factors with current asthma (such as obesity, having family members with asthma, passive smoke, the presence of mould or moisture in the home, and the presence of cockroaches). The prevalence of suspected asthma varied according to marital status and appeared to be higher among single, divorced, and widowed people. The result is in keeping with the results of the Health and Social Protection Survey (ESPS) conducted by IRDES in 2006 which found a prevalence of current asthma of 7.9% (6.8–9.0) among people living alone compared to 6.1% (5.3–6.9) among couples with no children, and 6.6% (6.1–7.1) among couples with children [14]. As with obesity, marital status is probably related to socioeconomic factors. In general, single-parent families tend to have a lower standard of living and are more likely to suffer from poverty.

Several environmental factors appear to be connected to the prevalence of suspected asthma (such as living close to a very busy road, a water treatment plant, or a source of harmful substances or air pollution). One particularly interesting factor identified in this study concerns the use of insecticide sprays at home, with more frequent use being associated with a higher prevalence of suspected asthma. Similar results can be found in the literature regarding the link between the use of home aerosolised cleaning products and asthma or other respiratory symptoms [34, 35]. This result is important in Reunion given the permanent risk posed by arboviruses outbreak and regular advice on preventing mosquitos’ bites [36, 37]. The result should be shared with health authorities in order to promote other methods of protection against insects (including mosquitoes) inside homes.

Our study has a number of limitations. The sampling methods excluded individuals with no landline or mobile phone, a group that makes up only a small portion (estimated at 3%) of the study population. The method used for selecting the interviewees partly consisted of repeating the random call back process to generate the required sample sizes for both categories of respondents (landline and only mobile); this may have introduced a selection bias insofar as the most available individuals were favoured for selection.

The study was conducted from the beginning of February to the end of May, outside the Austral winter (June to September) which may have led to an underestimation of the prevalence as individuals might have been more inclined to report asthma symptoms during the colder months. However, the questionnaire asked about asthma attacks, treatments or symptoms experienced in the preceding 12 months, thereby including the Austral winter period.

The prevalence estimates do not rely on a confirmed medical diagnosis including pulmonary function tests, but rather on self-reported symptoms or the prescription of medical treatment for asthma. For this reason, the classification is subject to possible reporting biases, especially regarding exposure to risk factors, which depends on the participants’ status and ability to recollect past events. In order to reduce the reporting bias for asthma, all questions relating to symptoms were asked before mentioning asthma itself. Since the profile of those who refused to participate and the reasons for non-participation are unknown, it was impossible to compare respondents and non-respondents to assess the impact of any non-response bias.

Furthermore, data about risk factors such as exposure to air pollution or allergens at home were not collected through objective measurements of air quality or allergy tests (such as skin prick tests or IgE blood tests), but rather relied on answers to questions about environmental characteristics.

Despite these limitations, this epidemiological study using standardised questionnaires and definitions comparable to those used in other national and international studies, enables us to compare the prevalence of asthma in Reunion with others published studies, and will be of value to health authorities and healthcare professionals.

## Conclusion

The results of this first survey on the prevalence of asthma in the adult population of Reunion Island provides novel information on the characteristics of asthmatic patients and on the environmental factors associated with diagnosed asthma and suspected asthma in this French territory.

One of the major findings of this study is the proportion of individuals with suspected asthma who have not been medically diagnosed with asthma and who are not receiving any treatment. Consequently, we recommend the development of a strategy in conjunction with general practitioners to improve the diagnosis of asthma on Reunion, based on the support of general practitioners. Ongoing care and therapeutic education of diagnosed persons is also needed to reduce the burden of the disease.

The results are also consistent with the literature regarding the links between asthma and environmental factors, such as the presence of moisture or cockroaches, and the use of insecticide sprays at home. These factors are important as exposure is common in tropical climates such as that in Reunion.

In terms of prevention, individual protection measures can be recommended, such as aerating and ventilating homes to reduce indoor moisture and mould growth, maintaining hygiene standards through cleaning, proper storage of foods to mitigate against insects or cockroaches, and limiting the use of spray insecticides and household cleaning sprays at home.

Metrological studies for air inside homes should be considered in cooperation with indoor environment medical advisers (a relatively new profession in Reunion) in order to improve our understanding of allergens present in domestic environments in tropical areas.

## Data Availability

The datasets used and/or analysed during the current study are available from the corresponding author on reasonable request.

## Abbreviations

BMI: Body Mass Index
CATI: Computer Assisted Telephone Interview
CCEP: Internal expert committee of The French Public Health Agency for project evaluation
CI: Confidence Interval
CNIL: National Commission on Informatics and Freedoms
COPD: Chronic Obstructive Pulmonary Disease
ECRHS: European Community Respiratory Health Survey
EGEA: Epidemiological study on the Genetics and Environment of Asthma
ESPS: Health and Social Protection Survey
INSEE: French National Institute of Statistics and Economic Studies
IRDES: Institute for Research and Documentation in Health Economics
OR: Odd Ratio
WHO: World Health Organisation

## References

[1] http://www.who.int/mediacentre/factsheets/fs307/en/. Date of access: April 10 2018.

[2] Delmas M-C, Furhman C (2010). L’asthme en France : synthèse des données épidémiologiques descriptives. Rev Mal Respir.

[3] Delmas M.-C., Guignon N., Leynaert B., Com-Ruelle L., Annesi-Maesano I., Herbet J.-B., Fuhrman C. (2009). Prévalence de l’asthme chez l’enfant en France. Archives de Pédiatrie.

[4] Solet J-L, Catteau C, Nartz E, Ronat J, Delmas M-C (2006). Epidémiologie de l’asthme à La Réunion : analyse de la mortalité (1990-1998) et de la morbidité hospitalière (1998-2002). Bull Epidemiol Hebd 2006.

[5] Fuhrman C, Nicolau J, Rey G, Solet J-L, Quenel P, Jougla E, et al. Asthme et BPCO : taux d’hospitalisation et de mortalité dans les départements d’outre-mer et en France métropolitaine, 2005-2007. Bull Epidemiol Hebd. 13(14):168–72.

[6] Prevot L, Dalleau-Passarelli N, Soulas A, Trevidic E (2003). Prise en charge médicale des patients asthmatiques : enquête de pratique auprès d’assurés sociaux et de médecins de la Réunion. Rev Med Ass Maladie.

[7] Martignon G, Catteau C, Debotte G, Duffaud B, Lebot F, Annesi-Maesano I (2004). Allergies infantiles à l’île de La Réunion : existe-t-il des différences avec la métropole ?. Rev Epidemiol Santé Publique.

[8] Delmas M-C, Guignon N, Leynaert B, Annesi-Maesano I, Com-Ruelle L, Gonzales L (2012). Prévalence et contrôle de l’asthme chez le jeune enfant en France. Rev Mal Respir.

[9] Burney PG, Luczynska C, Chinn S, Jarvis D (1994). The European Community respiratory health survey. Eur Respir J.

[10] Kauffmann F, Annesi-Maesano I, Liard R, Paty E, Faraldo B, Neukirch F (2002). Construction et validation d’un questionnaire en épidémiologie respiratoire. L’exemple du questionnaire de l’Etude Epidémiologique des facteurs Génétiques et Environnementaux de l’Asthme, l’hyperréactivité bronchique et l’atopie (EGEA). Rev Mal Respir.

[11] Equipements en communications électroniques et audiovisuels des ménages et des individus La Réunion In: l’Autorité de Régulation de Communication Electronique et des Postes. Site disponible sur: https://archives.arcep.fr/uploads/tx_gspublication/etude-equipements-usages-2012-Reunion-juil2013.pdf.

[12] Sistek D, Tschopp JM, Schindler C, Brutsche M, Ackermann-Liebrich U, Perruchoud AP (2001). Clinical diagnosis of current asthma: predictive value of respiratory symptoms in the SAPALDIA study. Swiss Study on Air Pollution and Lung Diseases in Adults. Eur Respir J.

[13] www.irdes.fr/donnees/556-enquete-sur-la-sante-et-la-protection-sociale-2012-etat-de-sante-1.xls. Date of access : November 10 2017.

[14] To (2012). Global asthma prevalence in adults: findings from the cross-sectional world health survey. BMC Public Health.

[15] Afrite A, Allonier C, Com-Ruelle L, Le Guen N (2011). L’asthme en 2006 : prévalence, contrôle et déterminants. IRDES.

[16] Moffatt MF, Gut IG, Demenais F, Strachan DP, Bouzigon E, Heath S (2010). A large-scale, consortium-based Genomewide association study of asthma. N Engl J Med.

[17] Boulet L-P (2013). Asthma and obesity. Clin Exp Allergy.

[18] Sutherland ER (2014). Linking obesity and asthma. Ann N Y Acad Sci.

[19] Trunk-Black Juel C, Suppli Ulrik C (2013). Obesity and asthma: impact on severity, asthma control, and response to therapy. Respir Care.

[20] Zock Jan-Paul, Jarvis Deborah, Luczynska Christina, Sunyer Jordi, Burney Peter (2002). Housing characteristics, reported mold exposure, and asthma in the European Community Respiratory Health Survey. Journal of Allergy and Clinical Immunology.

[21] Sharpe RA, Bearman N, Thornton CR, Husk K, Osborne NJ (2015). Indoor fungal diversity and asthma: a meta-analysis and systematic review of risk factors. J Allergy Clin Immunol.

[22] Mendell MJ, Mirer AG, Cheung K, Tong M, Douwes J (2011). Respiratory and allergic health effects of dampness, mold, and dampness-related agents: a review of the epidemiologic evidence. Environ Health Perspect.

[23] Kilpelainen M, Terho EO, Helenius H, Koskenvuo M (2001). Home dampness, current allergic diseases, and respiratory infections among young adults. Thorax.

[24] Evans J, Hyndman S, Stewart-Brown S, Smith D, Petersen S (2000). An epidemiological study of the relative importance of damp housing in relation to adult health. J Epidemiol Community Health.

[25] Williamson IJ, Martin CJ, McGill G, Monie RDH, Fennerty AG (1997). Damp housing and asthma: a case-control study. Thorax.

[26] Pirhonen I, Nevalainen A, Husman T, Pekkanen J (1996). Home dampness, moulds and their influence on respiratory infections and symptoms in adults in Finland. Eur Respir J.

[27] Brunekreef B (1992). Damp housing and adult respiratory symptoms. Allergy.

[28] Dales RE, Burnett R, Zwanenburg H (1991). Adverse health effects among adults exposed to home dampness and molds. Am Rev Respir Dis.

[29] Platt SD, Martin CJ, Hunt SM, Lewis CW (1989). Damp housing, mould growth, and symptomatic health state. BMJ.

[30] Arruda LK, Vailes LD, Ferriani VP, Santos AB, Pomés A, Chapman MD (2001). Cockroach allergens and asthma. J Allergy Clin Immunol.

[31] Arruda LK, Chapman MD (2001). The role of cockroach allergens in asthma. Curr Opin Pulm Med.

[32] Sastre J, Ibanet MD, Lombardero M, Laso MT, Lehrer S (1996). Allergy to cockroaches in patients with asthma and rhinitis in an urban area (Madrid). Allergy.

[33] Fraser BN (1979). Cockroaches in relation to bronchial asthma in the Durban area. S Afr Med J.

[34] Le Moual N, Varraso R, Siroux V, Dumas O, Nadif R, Pin I (2012). Domestic use of cleaning sprays and asthma activity in females. Eur Respir J.

[35] Bédard A, Varraso R, Sanchez M, Clavel-Chapelon F, Zock J-P, Kauffmann F (2014). Cleaning sprays, household help and asthma among elderly women. Respir Med.

[36] D’Ortenzio E, Balleydier E, Baville M, Filleul L, Renault P. Dengue fever in the Reunion Island and in South Western islands of the Indian Ocean. Med Mal Infect. 2011. 10.1016/j.medmal.2010.11.021 21295427

[37] Renault P, Solet JL, Sissoko D, Balleydier E, Larrieu S (2007). Filleul, et al. a major epidemic of chikungunya virus infection on Reunion Island, France, 2005–2006. Am J Trop Med Hyg.

